# The Modified Trochleoplasty for Trochlear Dysplasia Types B and D

**DOI:** 10.1016/j.eats.2025.103655

**Published:** 2025-05-22

**Authors:** Sergio Marinho de Gusmão Canuto, Arthur Macedo de Gusmão Canuto, Camilo Partezani Helito, Pedro Baches Jorge, Vitor Barion Castro de Padua, Diego Ariel de Lima

**Affiliations:** a Brazilian Storm Knee Research Group (BS Knee), São Paulo, Brazil; b Ortoclinica, Hospital de Ortopedia, Maceió, Brazil; c Universidade de São Paulo (USP), Grupo de Joelho, Instituto de Ortopedia e Traumatologia, Hospital das Clínicas, Faculdade de Medicina da Universidade de São Paulo (HCFMUSP), São Paulo, Brazil; d Santa Casa de Misericordia de São Paulo, São Paulo, Brazil; e Universidade de Marília (UNIMAR), Marília, Brazil; f Universidade Federal Rural do Semi-Árido (UFERSA), Mossoró, Brazil

## Abstract

Trochlear dysplasia is a major risk factor for recurrent patellar instability, often requiring surgical intervention in high-grade cases. The present modified trochleoplasty technique, known as the “Brazilian trochleoplasty,” is a reproducible technique developed by the Brazilian Storm Knee Research Group, which reshapes the trochlear groove while preserving cartilage integrity. Our technique is indicated for patients with recurrent patellar dislocation; abnormal patellar tracking, such as the J-sign; and high-grade trochlear dysplasia classified as Dejour types B and D, with trochlear prominence greater than 5 mm. This approach creates a single central sulcus without additional osteotomies, simplifying the procedure while ensuring anatomic precision. Fixation is achieved using headless compression screws, enhancing stability while minimizing cartilage damage. Compared with traditional sulcus-deepening techniques, the modified trochleoplasty offers a more accessible and effective alternative, improving patellar stability and reducing complications such as stiffness. Its stepwise methodology facilitates reproducibility, making it a viable option for young, active patients with severe trochlear dysplasia.

Trochlear dysplasia is a well-recognized anatomic abnormality and a significant risk factor for recurrent patellar instability.^1^ It is characterized by an abnormal shape of the trochlear groove, which compromises the engagement of the patella during knee flexion. This condition is particularly relevant in young, active individuals because it predisposes them to repeated dislocations, pain, and eventual degeneration of the patellofemoral joint.^2^ The management of trochlear dysplasia often involves surgical intervention, particularly in high-grade cases in which conservative measures fail to provide satisfactory outcomes.^2^, ^3^, ^4^

The classification of trochlear dysplasia proposed by Dejour et al.^1^ remains the cornerstone for assessing its severity and guiding treatment. Trochlear dysplasia is categorized into 4 types, each with distinct radiographic and morphologic features: type A, in which the trochlea is shallow but symmetrical and concave; type B, which is characterized by a supratrochlear spur and a flat or convex trochlea on axial imaging; type C, which presents a double-contour sign due to medial facet hypoplasia and a convex lateral facet; and type D, the most severe form, which combines the crossing sign, a supratrochlear spur, and the double contour sign extending below the crossing sign, with a “cliff-like” pattern on axial imaging. This classification aids in guiding diagnosis and surgical management.^5^^,^^6^

This article describes a technique for treating trochlear dysplasia types B and D, known as the “Brazilian trochleoplasty,” developed by the Brazilian Storm Knee Research Group. This approach aims to provide an effective and reproducible method for reshaping the trochlear groove, improving patellar stability, and minimizing complications while adhering to the anatomic and biomechanical principles essential for long-term joint preservation.

## Surgical Technique

The complete technique is demonstrated in Video 1. Pearls and pitfalls are presented in Table 1, and advantages and disadvantages are described in Table 2.

**Table 1:** Pearls and Pitfalls

Pearls
Precise preoperative planning should be performed with radiographic evaluation (Dejour classification, trochlear prominence, TT-TG distance) to determine indications.
The new trochlear groove position should be marked before bone resection to ensure anatomic alignment.
Preservation of a distal bone hinge allows controlled trochlear reshaping while maintaining stability.
Use of headless compression screws minimizes cartilage damage and provides stable fixation.
Systematic MPFL reconstruction optimizes patellar stability postoperatively.
Postoperative early mobilization reduces the risk of stiffness and improves functional outcomes.
Pitfalls
Inadequate patient selection (e.g., presence of advanced patellofemoral osteoarthritis) may lead to poor outcomes.
Excessive bone removal may compromise trochlear integrity and lead to iatrogenic instability.
Insufficient distal bone preservation can lead to trochlear collapse and loss of concavity.
Poor screw placement may lead to inadequate fixation, resulting in loss of trochlear shape or early failure.
Failure to assess associated patellar malalignment (e.g., high TT-TG distance) may lead to persistent instability.
Delayed rehabilitation or excessive restriction of movement may result in arthrofibrosis.

MPFL, medial patellofemoral ligament; TT-TG, tibial tubercle–trochlear groove.

**Table 2:** Advantages and Disadvantages

Advantages
This technique preserves cartilage integrity while effectively reshaping the trochlea.
This technique is less invasive than traditional deepening trochleoplasty techniques requiring multiple osteotomies.
Headless compression screws are used, reducing the risk of cartilage injury compared with anchors or headed screws.
A single, anatomically correct central sulcus is created, simplifying the procedure.
Systematic MPFL reconstruction enhances patellar stability.
The stepwise methodology of this technique improves reproducibility and facilitates implementation.
Disadvantages
The procedure is technically demanding with a learning curve for proper execution.
Precise bone removal is required to avoid overcorrection or insufficient trochlear reshaping.
There is a risk of intraoperative trochlear fracture if excessive force is applied during reshaping.
Limited long-term outcome data are available compared with established sulcus-deepening techniques.
This technique is not indicated for patients with advanced patellofemoral osteoarthritis.
Careful preoperative assessment is required to determine appropriate candidates.

MPFL, medial patellofemoral ligament.

### Surgical Indications

**Fig 1:**
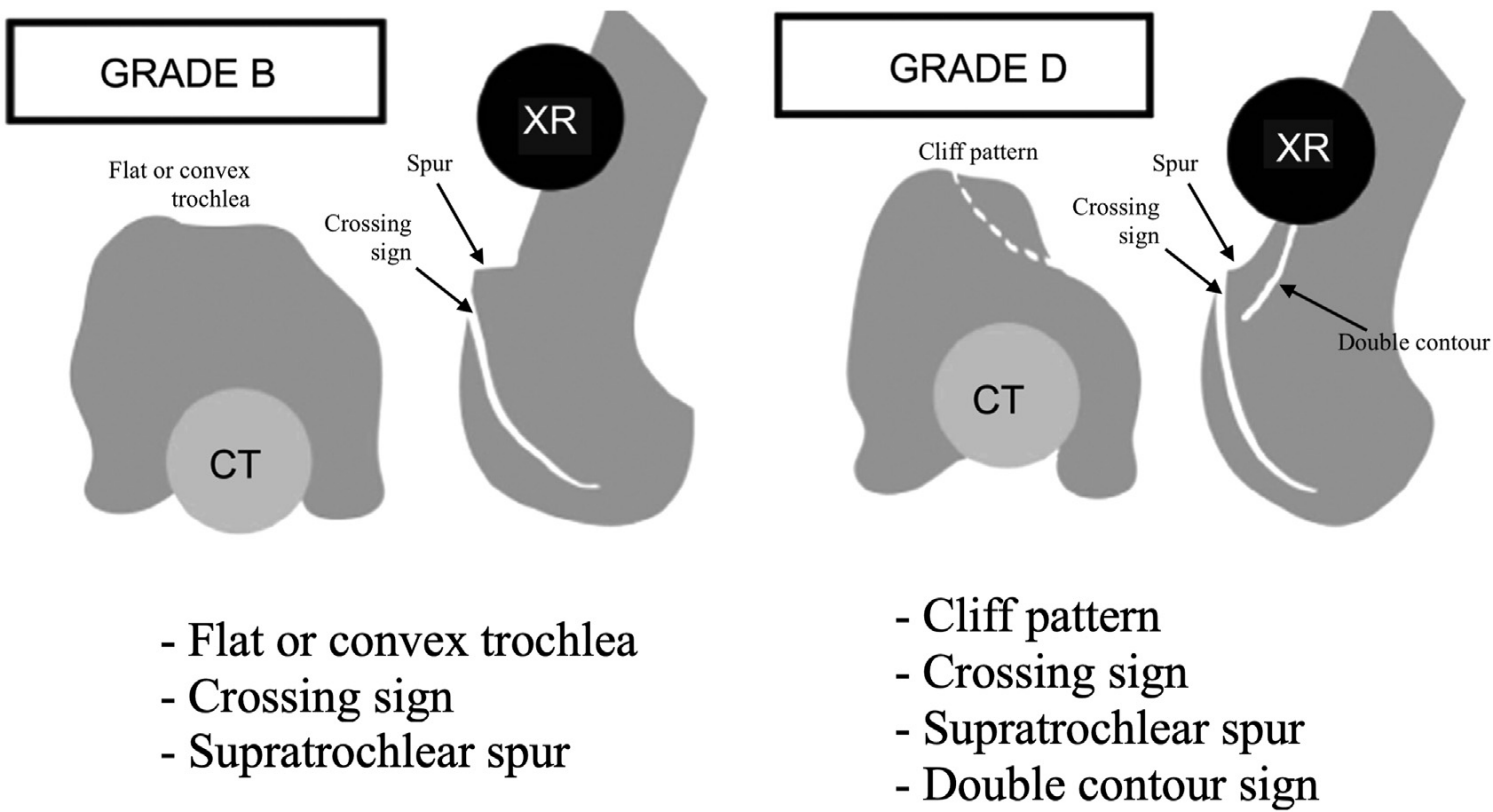
High-grade trochlear dysplasia classified as type B or D according to Dejour et al.^1^ Type B is characterized by a supratrochlear spur and a flat or convex trochlea on axial imaging. Type D, the most severe form, combines the crossing sign, a supratrochlear spur, and the double contour sign extending below the crossing sign, with a cliff-like pattern on axial imaging. The axial images were obtained from computed tomography (CT), and the lateral views were obtained from conventional radiographs (XR) (Right knee).

**Fig 2:**
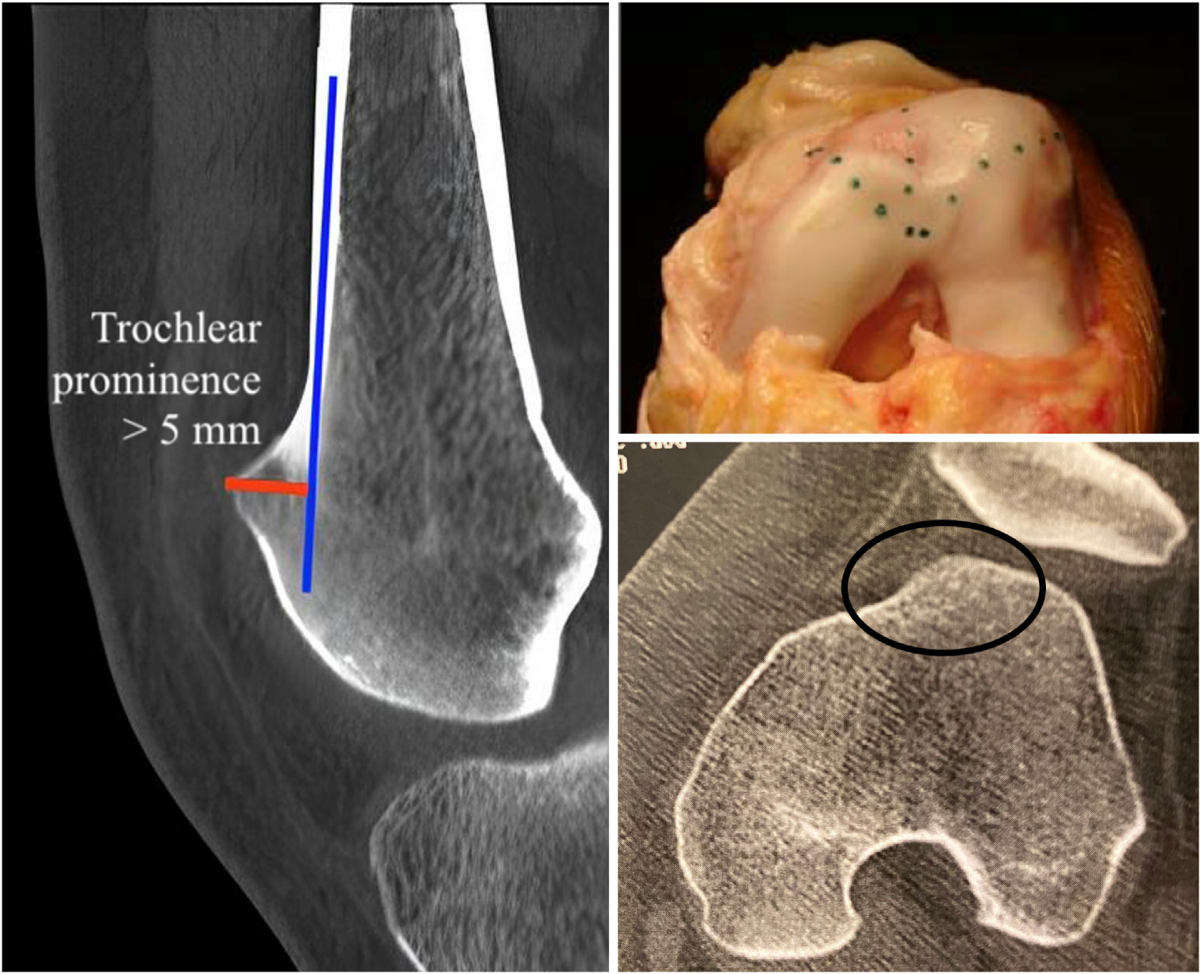
Trochlear prominence greater than 5 mm, as seen on computed tomography in sagittal and axial views, and during surgery (left knee). Blue line indicates anterior cortical and orange oval lines indicate troclear proeminence.

Our technique is indicated for patients with recurrent patellar dislocation; abnormal patellar tracking, such as the J-sign; and high-grade trochlear dysplasia classified as type B or D (Fig 1) according to Dejour et al.,^1^ with trochlear prominence greater than 5 mm (Fig 2).^4^^,^^7^ Contraindications include patellofemoral arthritis or high-grade trochlear cartilage wear, isolated patellofemoral pain without true episodes of patellar dislocation, and skeletally immature patients.^8^ Additionally, patients with patellar instability who lack trochlear dysplasia or have low-grade Dejour type C and/or A dysplasia (dysplasia with an almost normal sulcus angle) are unlikely to benefit from our technique.^8^

### Necessary Materials for the Procedure

The following materials are required for the procedure: two headless compression screws, such as the Acutrak Headless Compression Screw System® (Acumed, Hillsboro, OR, USA), MAX VPC Screw System® (Zimmer Biomet, Warsaw, IN, USA), Compression FT Screw® (Arthrex, Naples, FL, USA), or REDUCT Headless Compression Screw (Skeletal Dynamics, Miami, FL, USA). Screws with continuously variable thread pitch are preferred over Herbert-type screws due to superior compression and stability. Additional equipment includes an orthopedic oscillating bone saw and, optionally, an orthopedic surgical burr for refinement, bone grinding, and cartilage surface preparation.

### Patient Positioning

Sedation and regional anesthesia are used for this procedure. The patient is placed in a supine position, with 2 supports (lateral and distal) used to maintain knee flexion. A high tourniquet is preferred, ensuring the administration of prophylactic intravenous antibiotics before tourniquet inflation. Tilt testing and patellar glide are evaluated.

### Surgical Approach

**Fig 3:**
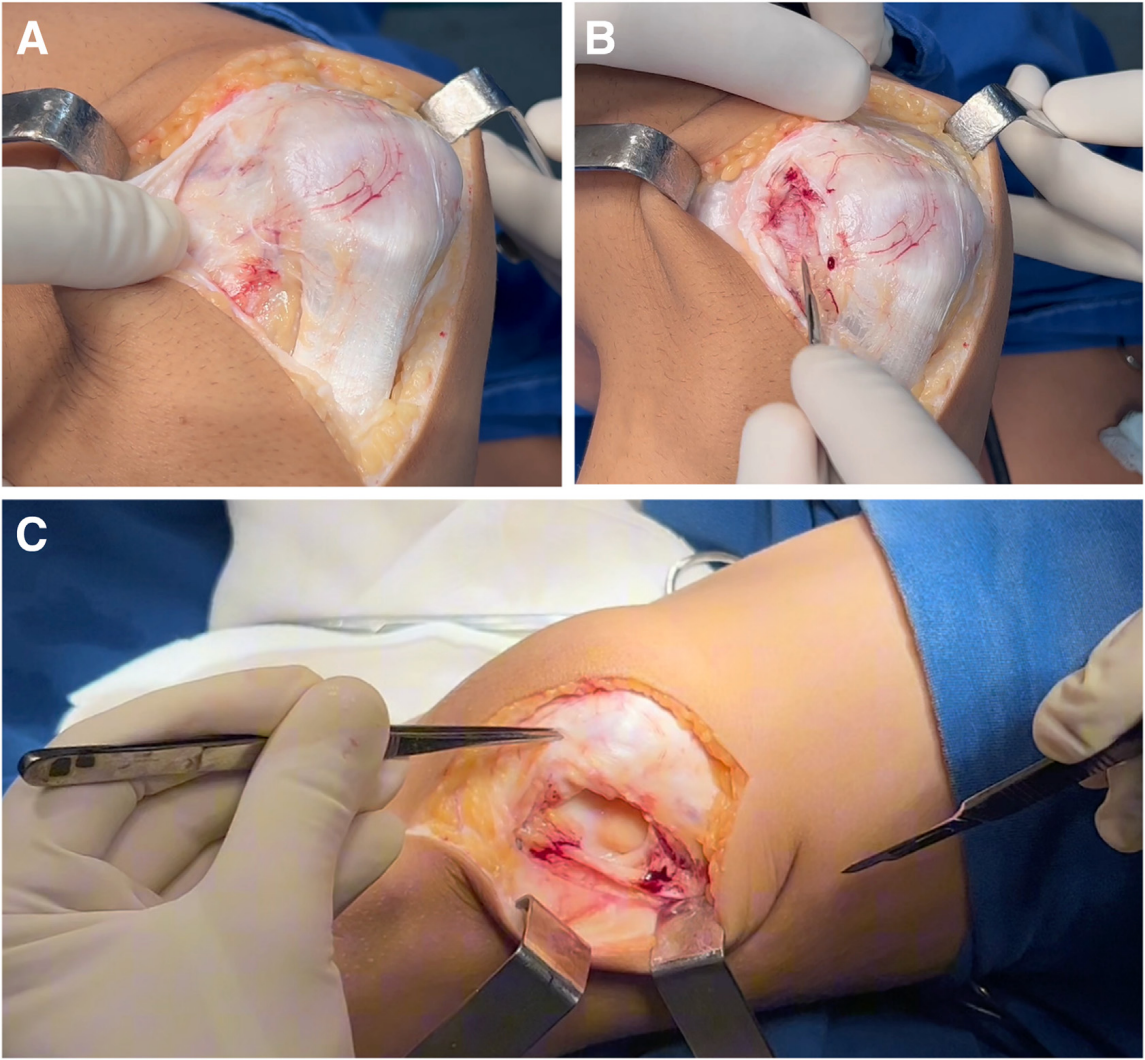
Surgical approach. (A) A 10- to 15-cm longitudinal midline incision is made over the knee. (B) Before the arthrotomy, the iliotibial tract is released. (C) The midvastus approach is used. For less experienced surgeons, a useful tip is to use the medial parapatellar arthrotomy because it facilitates exposure of the trochlea (right knee).

A longitudinal midline incision of 10 to 15 cm is made over the knee. Before the arthrotomy is performed, the iliotibial band is released, followed by cauterization of the vessels. The procedure then proceeds using the midvastus approach (for less experienced surgeons, a useful tip is to use the medial parapatellar arthrotomy because it facilitates exposure of the trochlea) (Fig 3). The periosteum of the medial third of the patella is removed to prepare it for subsequent medial patellofemoral ligament (MPFL) reconstruction, which is performed systematically.

The anterior surface of the distal femur serves as a reference to determine the amount of bone to be removed during trochleoplasty, ensuring the new groove is leveled with it. Trochlear dysplasia is then assessed by measuring the supratrochlear spur and frequently evaluating a hypoplastic medial facet. Chondral lesions are commonly found in type B or D dysplasia, with a clear correlation between trochlear dysplasia and cartilage defects.

### Trochlear Groove and Tibial Tubercle–Trochlear Groove Distance

The native groove and the boundaries of the lateral and medial facets are identified. Frequently, the native groove is abnormally displaced medially. Groove asymmetry is always present in type D dysplasia. Lateralization of the trochlear groove reduces the tibial tubercle–trochlear groove (TT-TG) distance.

**Fig 4:**
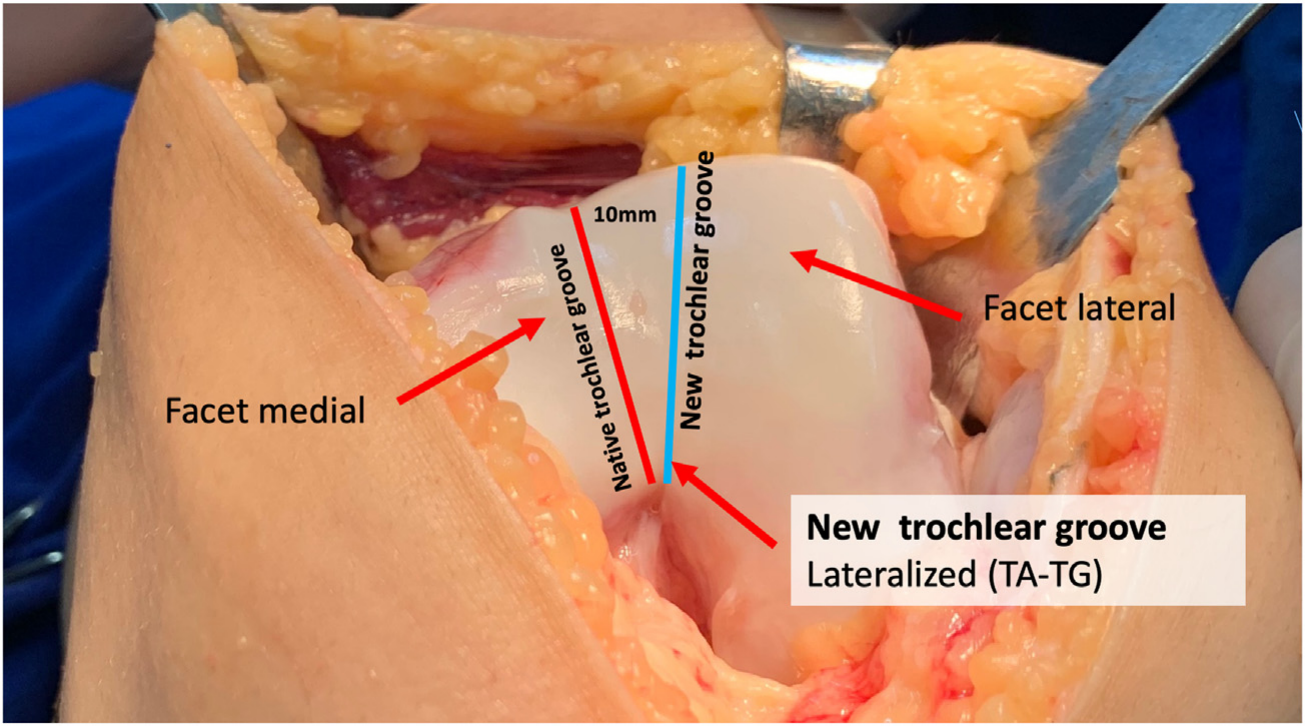
The planned new groove is marked in a more lateral position, ensuring alignment with the anatomic axis of the femur. Frequently, the native groove is abnormally displaced medially. This lateralized new groove position reduces the tibial tubercle–trochlear groove distance (left knee). TA-TG (TT-TG), tibial tubercle-trochlear groove distance.

**Fig 5:**
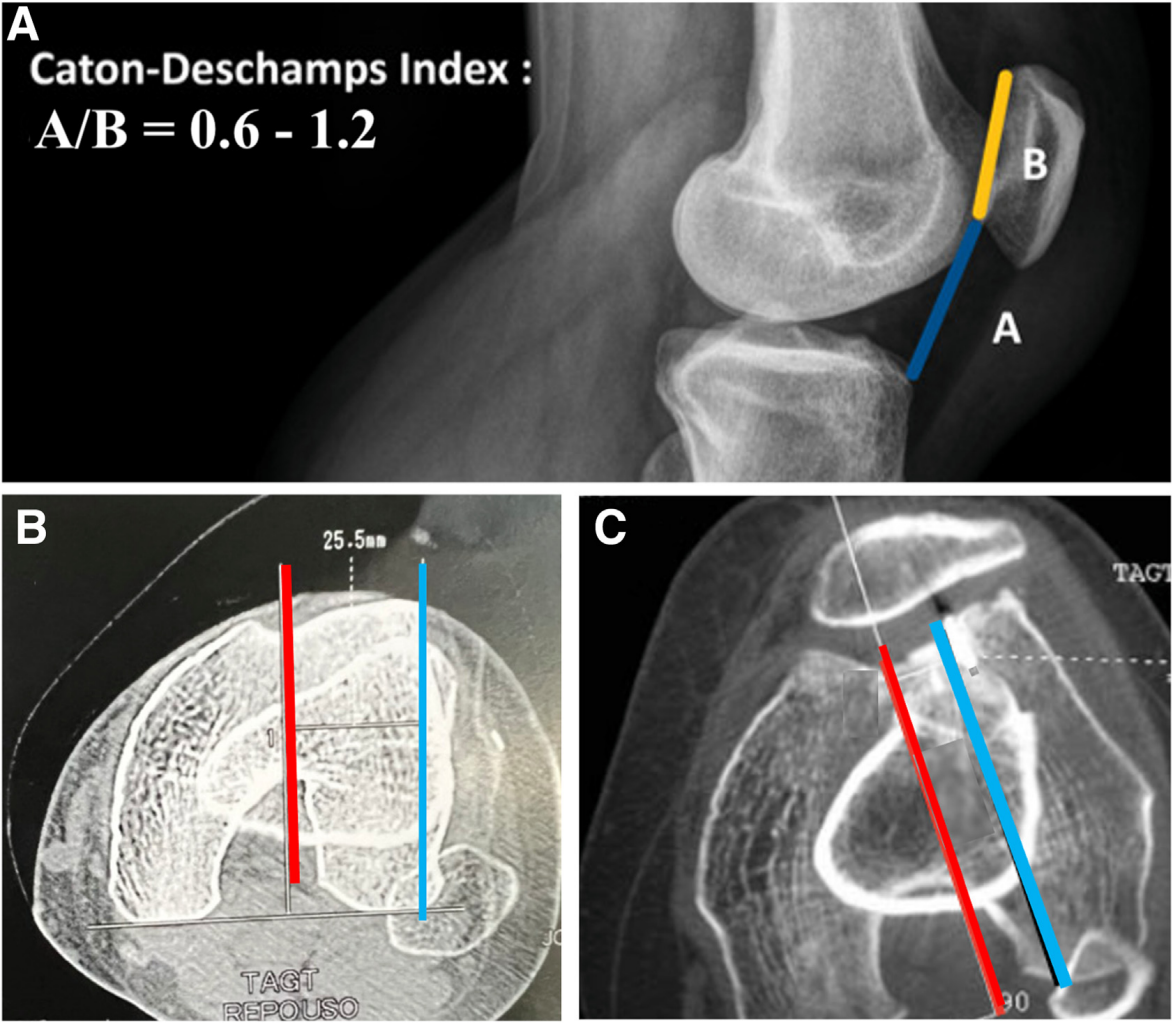
Tibial tubercle medialization and/or distalization osteotomy is indicated in association with our modified trochleoplasty technique if, after groove lateralization, the Caton-Deschamps index exceeds 1.2 (A) and/or the tibial tubercle–trochlear groove distance remains greater than 20 mm (B, C) (left knee). (A) Blue line (marked A) indicates distace between the inferior pole of the patella and the anterior edge of the tibial plateau. Yellow line (marked B) indicates length of the articular surface of the patella. (B, C) Red line indicates perpendicular line to the posterior condylar axis passing through the center of the trochlear groove. Blue line indicates perpendicular line to the posterior condylar axis passing through the center of the tibial tubercle.

The planned new groove is marked in a more lateral position, ensuring alignment with the anatomic axis of the femur (Fig 4). This lateralized groove position reduces the TT-TG distance. The distance between the native groove and the new groove is measured to assess the modification of the TT-TG distance. If, after groove lateralization, the TT-TG distance remains greater than 20 mm and/or the Caton-Deschamps index exceeds 1.2 (Fig 5), an associated tibial tubercle medialization and/or distalization osteotomy is performed.

### Removal of Trochlear Prominence

**Fig 6:**
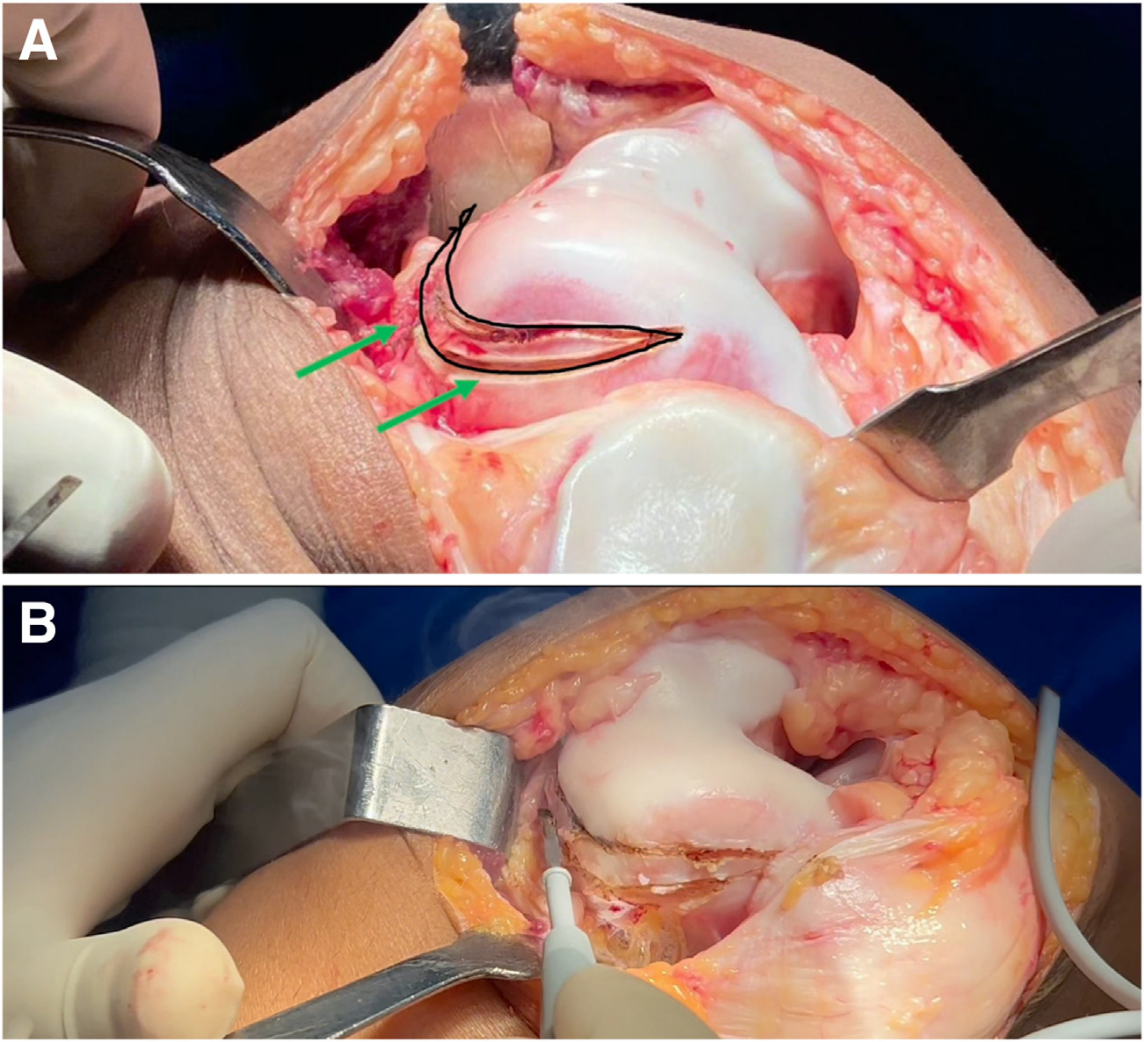
Removal of trochlear prominence. (A, B) The area of bone to be removed is marked with an electrocautery device (black outline and green arrows). The most critical step of the procedure is the elimination of the lateral trochlear prominence. A cortical bone wedge is excised at the lateral femoral-trochlear osteochondral junction (right knee).

The most critical step of the procedure is the elimination of the lateral trochlear prominence. A cortical bone wedge is excised at the lateral femoral-trochlear osteochondral junction. The area to be resected is marked using an electrocautery device (Fig 6), and the osteotomy is performed with an oscillating saw. The thickness of the resected wedge corresponds to the height of the trochlear prominence, allowing the new trochlear groove to align flush with the anterior cortex of the femur.

The volume of bone resection varies according to the severity of trochlear dysplasia and the degree of realignment required. The objective is to eliminate the prominence while creating an anatomically congruent groove aligned with the anterior femoral cortex, typically preserving a residual bone thickness of approximately 4 mm beneath the trochlear cartilage. The resection usually follows a triangular configuration, with a broad proximal base and progressive depth extending distally.

**Fig 7:**
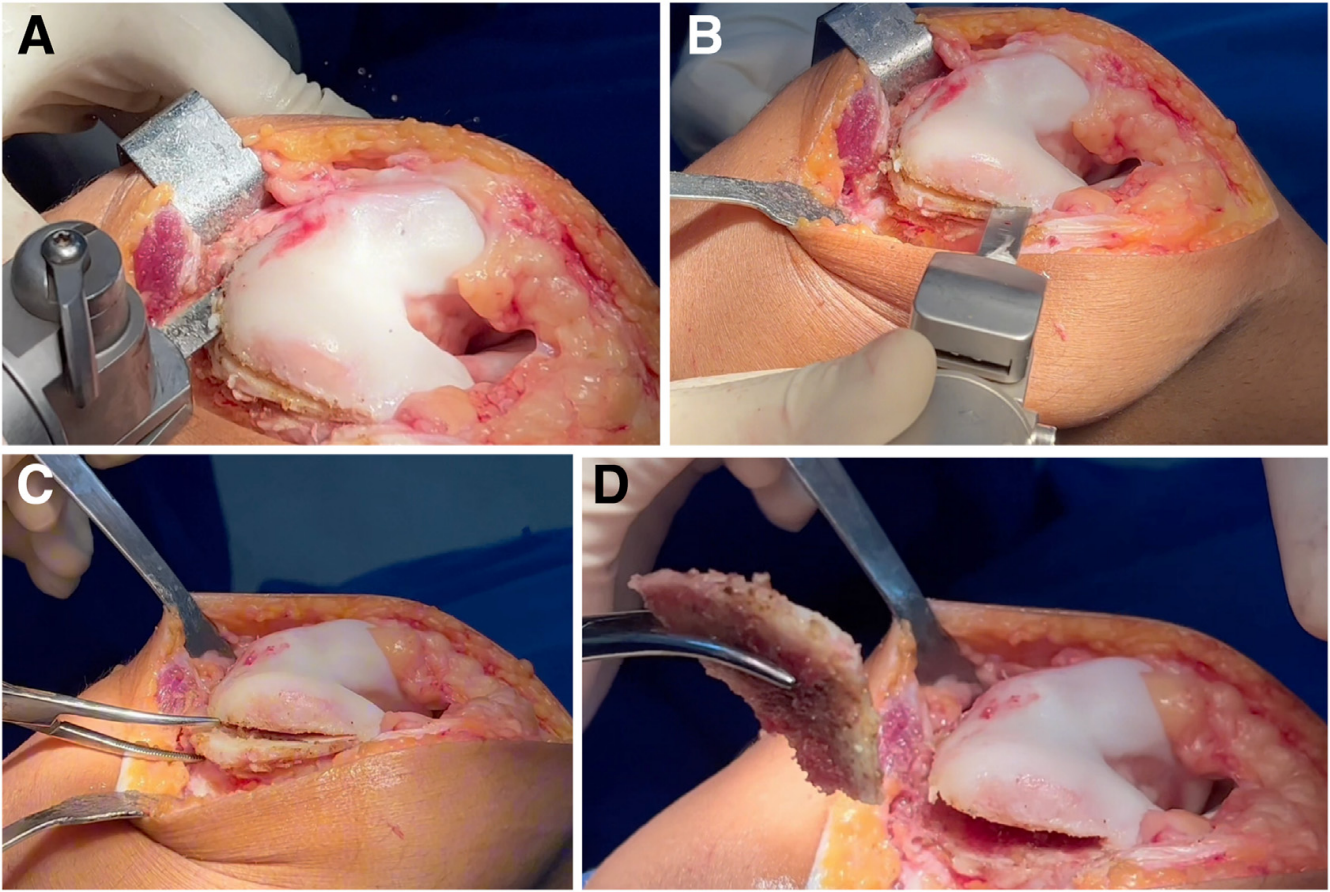
Removal of trochlear prominence. (A, B) An oscillating saw is used for the osteotomy. (C, D) Typically, bone removal follows a triangular pattern, with a proximal base and variable depth from proximal to distal (right knee).

In addition to the lateral femoral condyle osteotomy for prominence removal, a complementary osteotomy is performed on the medial and proximal trochlea. The amount of bone resected medially is generally less than that removed laterally, where the dysplasia is more pronounced. This difference accounts for the anatomic asymmetry and the need to preserve medial patellar stability. Medial bone removal is minimal and intended solely to facilitate the proper orientation of the new groove in relation to the anterior femoral cortex (Fig 7). If necessary, an orthopaedic surgical burr is used for refinement, contouring, and bone shaving. Spongy bone removal is performed in the metaphyseal region of the femur, aiming to preserve the trochlear thickness as much as possible on the medial side, particularly given that the medial condyle is hypoplastic in many cases.

### Trochlear Osteotomy: New Groove

A large surgical scalpel blade, preferably a No. 23 blade, is used to incise the cartilage along the previously marked path of the new groove. A mallet may be used in conjunction with the scalpel to facilitate the incision. If necessary, the groove can be further defined using a fine osteotome.

**Fig 8:**
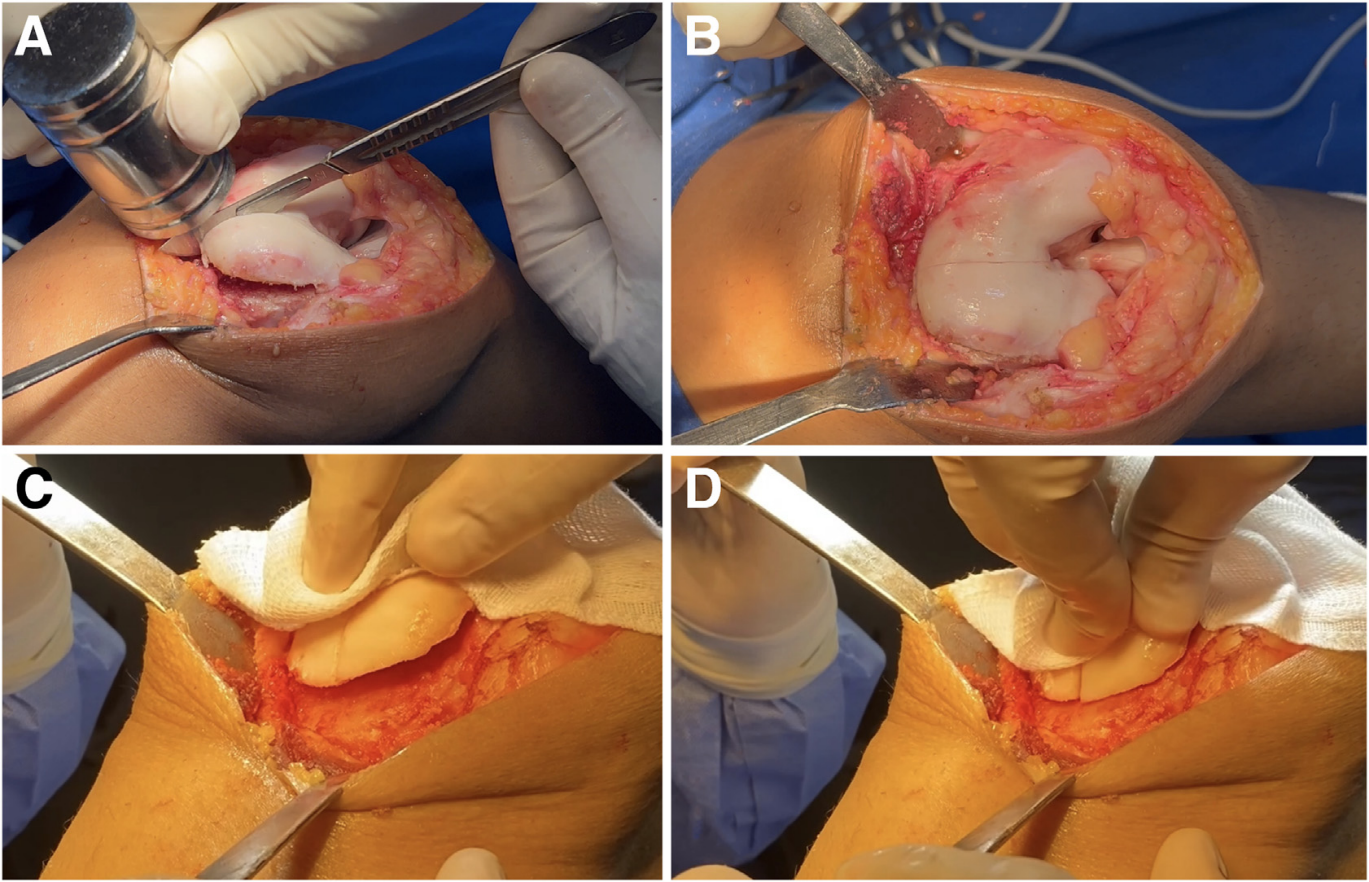
Trochlear osteotomy: new groove. (A, B) A surgical scalpel blade is used to incise the cartilage, positioning it along the previously marked new groove. A mallet may be used in conjunction with the scalpel to facilitate the incision. If necessary, the groove can be further defined using a fine osteotome. (C, D) At this stage, the entire trochlea can be mobilized by manual depression (right knee).

**Fig 9:**
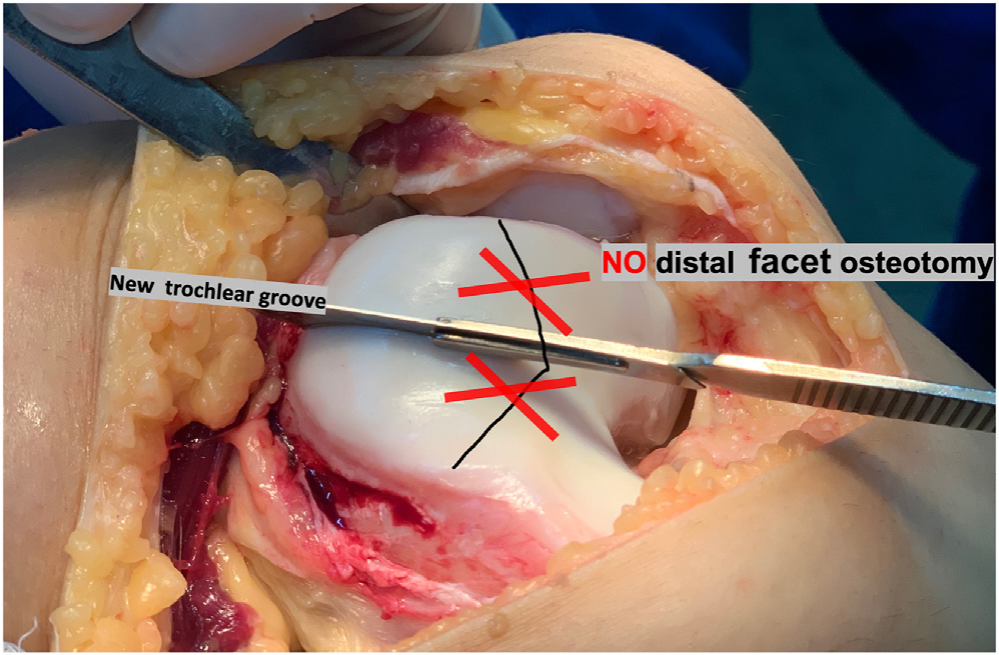
No distal facet osteotomy, preserving a distal hinge that maintains structural integrity. If the osteochondral flap is not sufficiently flexible to be molded into the desired contour without risk of fracture, additional distal cancellous bone should be carefully removed using a burr, oscillating saw, or curette. It is essential to preserve a distal hinge of bone to maintain the structural integrity of the flap. This step requires extreme caution to avoid compromising the continuity of the osteochondral segment (right knee). Red Xs and black line indicates distal limit of the osteotomy.

At this stage, the entire trochlear surface can be mobilized through manual depression (Fig 8). If the osteochondral flap is not sufficiently flexible to be molded into the desired contour without risk of fracture, additional distal cancellous bone should be carefully removed using a burr, oscillating saw, or curette. It is essential to preserve a distal “hinge” of bone to maintain the structural integrity of the flap. This step requires extreme caution to avoid compromising the continuity of the osteochondral segment (Fig 9).

The facets are manually pressed to verify that the new groove is level with the anterior femoral cortex. Some of the bone removed at the beginning of the procedure can be placed peripherally beneath the facets, helping to support the elevated flap position. Deepening trochleoplasty aims to reshape the dysplastic trochlea to better match patellar morphology, rather than creating a patellofemoral mismatch. Once the new groove is satisfactory and perfectly leveled, fixation is performed.

### Fixation

Fixation is performed using a simple technique with screws—one in each facet. The facets are pressed to ensure adequate compression, and guidewires are used for stabilization. The new groove must be aligned with the anterior femoral cortex.

**Fig 10:**
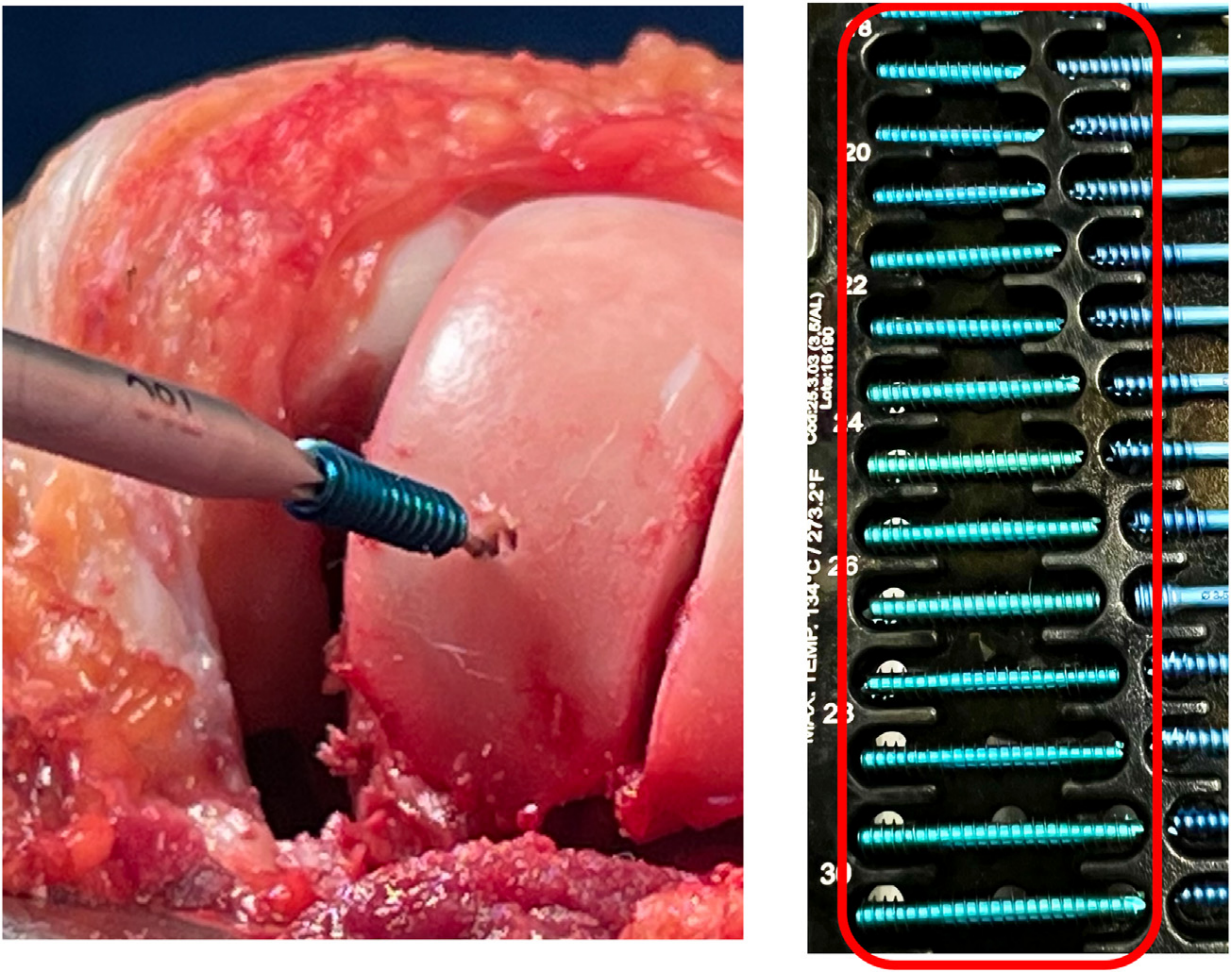
Headless compression screws (e.g., Acutrak Headless Compression Screw System, MAX VPC Screw System, Compression FT Screw, or REDUCT Headless Compression Screw). We avoid Herbert-style screws and prefer those with a continuously variable thread pitch (right knee).

**Fig 11:**
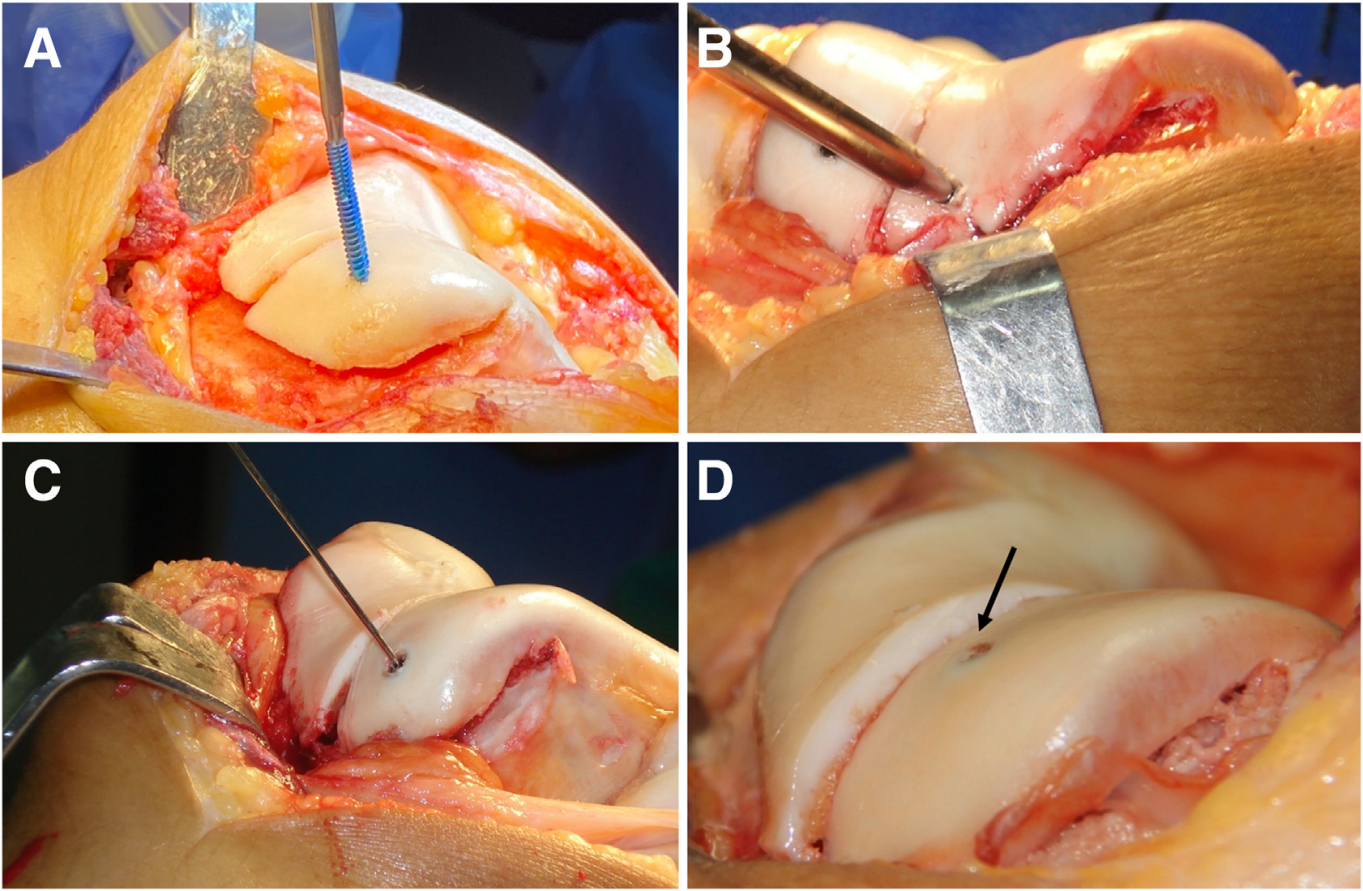
(A-D) The facets are pressed to ensure adequate compression, and the new groove must be aligned with the anterior femoral cortex. Once the new groove is satisfactory and perfectly leveled, fixation is performed. Fixation is achieved using two 3-mm headless compression screws, one medial and one lateral (arrow) (right knee).

Fixation is achieved using two 3-mm headless compression screws (Fig 10), one medial and one lateral (Fig 11). The use of Herbert-type screws is avoided; instead, screws with a continuously variable thread pitch are preferred, providing better compression and bone stability. This type of screw fixation minimizes cartilage surface damage compared with anchors or headed screws. After trochlear fixation, a well-corrected concavity is observed. The previously removed bone wedge can be fragmented and used as a graft to fill the spaces between the facets.

**Fig 12:**
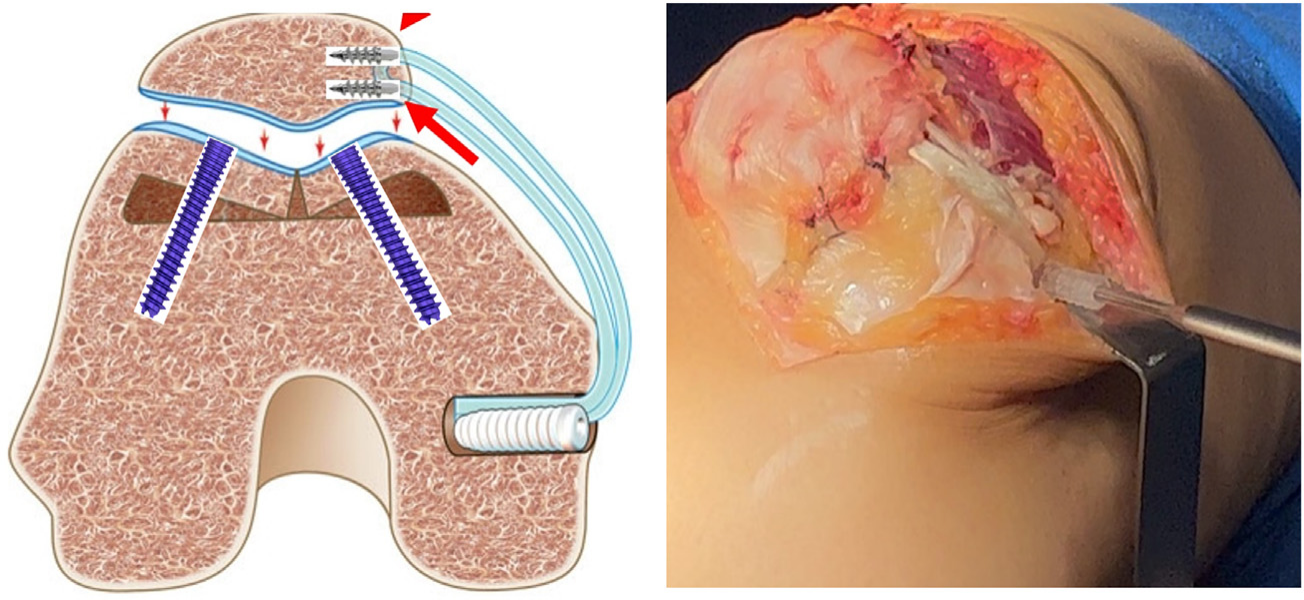
Medial patellofemoral ligament reconstruction is performed systematically at the end of the procedure (arrows) (right knee).

**Fig 13:**
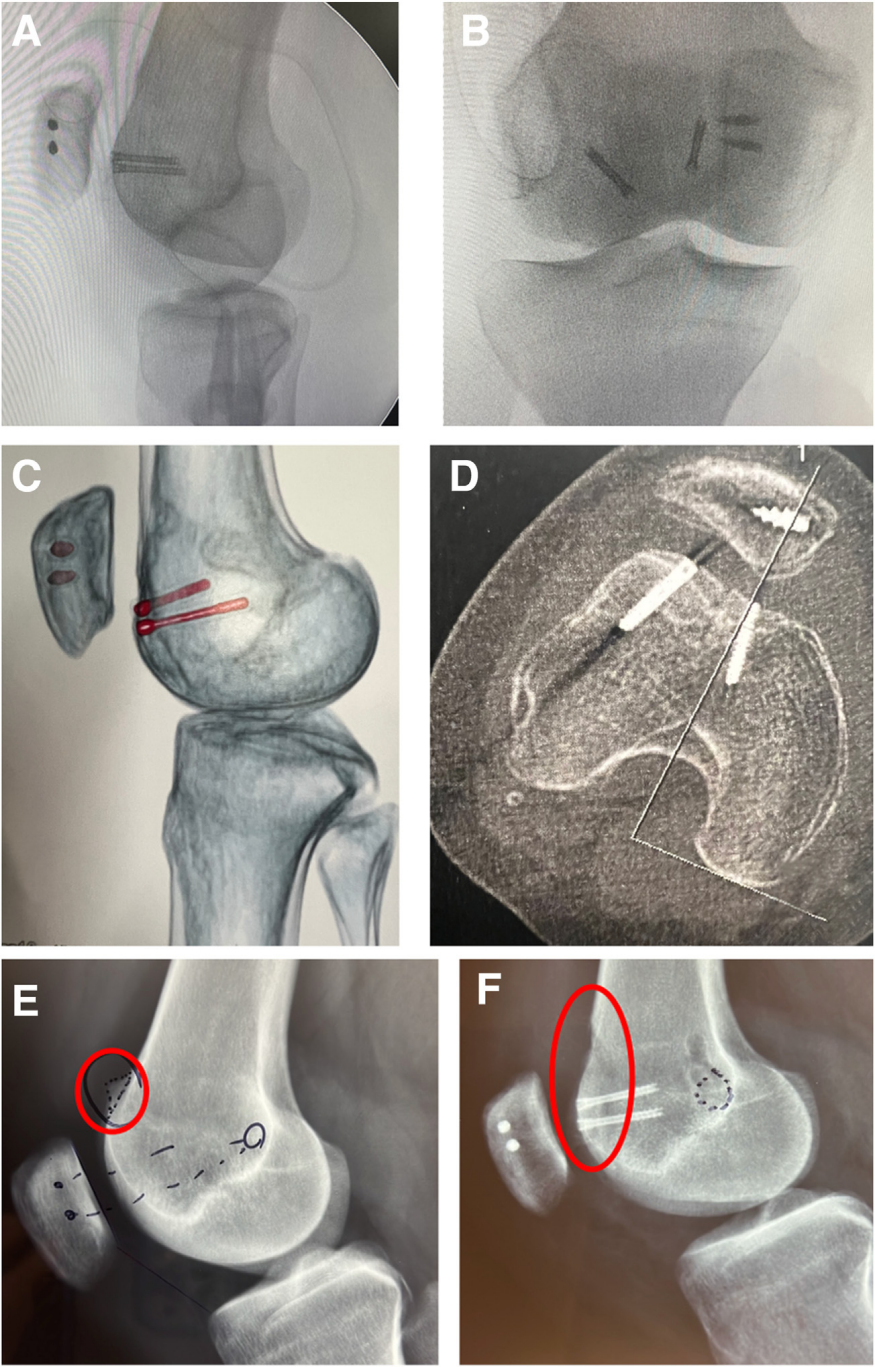
Imaging of modified trochleoplasty. (A, B) Postoperative control fluoroscopy. (C, D) Postoperative computed tomography scan. (E, F) Preoperative and postoperative radiographs (right knee). Oval indicates preoperative trochlear proeminence (E). Oval indicates postoperative trochlear proeminence (F).

Patellar tracking is tested before proceeding with tibial tubercle osteotomy and/or MPFL reconstruction. MPFL reconstruction is performed systematically at the end of the procedure (Fig 12). Layered closure is performed, and suction drainage is not used (Fig 13).

### Rehabilitation

Partial weight bearing is permitted as tolerated. After 4 weeks, full weight bearing in extension is allowed, with an emphasis on early range-of-motion exercises to prevent arthrofibrosis. In cases in which an associated tibial tubercle osteotomy was necessary, patients remain non–weight bearing for 4 weeks, followed by partial weight bearing for 2 weeks, until osteotomy consolidation is achieved.

## Discussion

Trochleoplasty, particularly via sulcus-deepening techniques, has evolved significantly since its inception. Traditional methods, such as those described by Dejour et al.^9^ and Bereiter and Gautier,^10^ aim to reshape the trochlear groove while preserving cartilage integrity and optimizing patellofemoral alignment. These procedures are technically demanding and require meticulous planning to avoid complications such as cartilage damage or postoperative stiffness. Despite the advances, there remains a need for techniques that balance effectiveness, reproducibility, and minimal invasiveness, particularly for high-grade dysplasia.^11^

Our technique simplifies the surgical approach by creating a single central sulcus without additional osteotomies. The use of headless compression screws ensures stable fixation while reducing costs, making the procedure more accessible and reproducible. By preserving cartilage integrity and optimizing trochlear morphology, this technique improves patellar stability and minimizes complications. Compared with traditional sulcus-deepening methods, this approach offers an effective, straightforward alternative for treating high-grade trochlear dysplasia.

## Disclosures

All authors (S.M.d.G.C., A.M.d.G.C., C.P.H., P.B.J., V.B.C.d.P., D.A.d.L.) declare that they have no known competing financial interests or personal relationships that could have appeared to influence the work reported in this paper.

## Supplementary Data

Video 1 Modified trochleoplasty for trochlear dysplasia types B and D: step-by-step guidance including approach, osteotomy, groove creation, fixation, and medial patellofemoral ligament (MPFL) reconstruction. Right knee.
